# Occurrence of *Enterobacteriaceae* in Raw Meat and in Human Samples from Egyptian Retail Sellers

**DOI:** 10.1155/2014/565671

**Published:** 2014-11-11

**Authors:** Mayada Gwida, Helmut Hotzel, Lutz Geue, Herbert Tomaso

**Affiliations:** ^1^Department of Hygiene and Zoonoses, Faculty of Veterinary Medicine, Mansoura University, Mansoura 35516, Egypt; ^2^Friedrich-Loeffler-Institut, Institute of Bacterial Infections and Zoonoses, 07743 Jena, Germany; ^3^Friedrich-Loeffler-Institut, Institute of Molecular Pathogenesis, 07743 Jena, Germany

## Abstract

The present study was performed to assess the presence of *Enterobacteriaceae* in raw meat and handlers in Egypt using cultivation and matrix-assisted laser desorption ionization time-of-flight mass spectrometry (MALDI-TOF MS). A total of 100 raw meat samples (chicken and beef meat, 50 each) were randomly purchased from butchers and local meat retailers located at Mansoura city, Egypt. Fifty human samples were collected from meat handlers (hand swabs and stool specimens, 25 each). 228 bacterial isolates were recovered from these samples. Unidentified isolates were characterized by partial 16S rRNA gene sequencing. *Escherichia coli* isolates were further typed using a DNA microarray system. *Proteus* spp. (60.0%) were found to be the most abundant followed by *Escherichia coli* (38.7%), *Klebsiella* spp. (17.3%), and *Citrobacter* spp. (13.3%). The presence of different *Enterobacteriaceae* in locally produced retail raw meat demonstrates the risk of infection of people through consumption of raw or undercooked meat and the risk for cross-contamination of other food products. Harmonized and concerted actions from veterinary and public health authorities are needed to reduce the risk of infection.

## 1. Introduction 

Most of the pathogens that play a role in foodborne diseases are of animal origin [1]. Foodborne diseases pose a serious threat to the health of people in Africa and cause huge economic losses [2]. Up to one-third of the population in developing countries is affected by foodborne diseases each year. It is assumed that foodborne and waterborne diarrheal diseases kill more than 2.2 million people each year [3]. A major problem in food hygiene is the fecal contamination of beef and chicken meat with* Enterobacteriaceae* such as* Salmonella* spp.,* Escherichia* (*E*.)* coli*,* Proteus* (*P*.), and* Klebsiella* (*K.*) species [4, 5]. To minimize the prevalence of foodborne diseases and to reduce the microbial contamination of food, effective monitoring of the occurrence and reliable identification of zoonotic bacterial pathogens in food is essential. Currently, routine detection of foodborne pathogens relies on cultivation and biochemical identification [6]. These methods are laborious and time-consuming, and may lead to false identifications [7]. In recent years, MALDI-TOF MS has been implemented in microbiological routine laboratories for broad-spectrum identification of bacteria [8–11]. The present study was performed to assess the presence of* Enterobacteriaceae* in raw meat and handlers in Egypt using cultivation of bacteria and MALDI-TOF MS. When MALDI-TOF MS lead to doubtful results, partial sequencing of 16S rRNA genes was used to verify the identification. Furthermore,* E. coli* isolates were characterized by microarray analysis.

## 2. Material and Methods

### 2.1. Sample Collection

The study was conducted between October 2012 and May 2013. A total of 100 fresh raw meat samples (chicken and beef meat, 50 each) were randomly purchased from butchers and local meat retailers located in Mansoura city, Egypt. In addition, 50 human samples (hand swabs and stool specimens from meat handlers, 25 each) were collected. The samples were placed on ice and transported immediately to the Hygiene and Zoonoses laboratory, Faculty of Veterinary Medicine, Mansoura University, Egypt. An informed consent was obtained from all persons involved in this study.

### 2.2. Sample Preparation

Twenty-five grams of each raw beef or chicken meat was transferred to a blender bag and homogenized with 225 mL of 0.1% buffered peptone water (BPW; Oxoid, Wesel, Germany). Pre-enrichment was done for 24 h at 37°C. A loop full of the enriched broth was streaked on MacConkey agar plates (Oxoid) and Eosin Methylene Blue Lactose plates (Oxoid) and incubated at 37°C for 24 h. For human samples, hand swabs and stool specimens were directly inserted into sterile tubes containing 10 mL BPW under aseptic conditions and incubated at 37°C for 24 h. Then, the samples were cultivated as described previously.

### 2.3. Bacterial Identification

Colonies were picked and streaked onto nutrient agar plates, incubated at 37°C for 18–24 h and then stored at −20°C as glycerol cultures until shipping to the Friedrich-Loeffler-Institut, Institute of Bacterial Infections and Zoonoses, Jena, Germany. In Germany, the bacterial isolates were identified using MALDI-TOF MS as described by Karger et al. [12]. Briefly, bacteria were cultivated on Columbia sheep blood agar at 37°C for 24 h. Single colonies were picked, suspended in 300 *μ*L of water, and precipitated by addition of 900 *μ*L of ethanol p. a. (Carl Roth GmbH, Karlsruhe, Germany). Samples were centrifuged at 10,000 g for two minutes. The supernatant was carefully removed and the sediment was resuspended in 50 *μ*L of 70% (v/v) formic acid (Sigma-Aldrich Chemie GmbH, Taufkirchen, Germany). After mixing with 50 *μ*L acetonitrile (Carl Roth GmbH), the suspension was centrifuged as described above and the supernatant was transferred into a fresh tube. One *μ*L of the supernatant was spotted two times onto a MALDI target plate (polished steel MTP 384 plate, Bruker Daltonik GmbH, Bremen, Germany) and allowed to dry at room temperature. Finally, the dried spots were overlaid with one *μ*L of matrix, which was a saturated solution of *α*-cyano-4-hydroxycinnamic acid (Sigma-Aldrich Chemie GmbH) in 50% acetonitrile and 2.5% trifluoroacetic acid (Sigma-Aldrich Chemie GmbH). As soon as the samples were air-dried-measurement was started within 10 min.

Spectra were acquired with 300 laser shots with an Ultraflex I instrument (Bruker Daltonik GmbH) in the linear positive mode in the range of 2,000 to 20,000 Da. Acceleration voltage was 25 kV and the instrument was calibrated in the range between 3,637.8 and 16,952.3 Da using the IVD Bacterial Test Standard Calibrant (BTS; Bruker Daltonik GmbH). For species identification, the BioTyper database 3.0 (Bruker Daltonik GmbH) was used. An identification (ID) score >2.30 is regarded a highly probable species identification; scores 2.0–2.29 indicate secure genus and probable species identification; scores 1.70–1.99 allow probable genus identification and lower scores provide no reliable results (Table 3). Unidentified bacteria and bacteria with a score <2.0 were identified using partial 16S rRNA gene sequencing.

### 2.4. DNA Extraction and Partial 16S rRNA Gene Sequencing

Isolation of DNA was carried out with a High Pure PCR Template Preparation Kit (Roche Diagnostics, Mannheim, Germany) according to the manufacturer's instructions. Partial 16S rRNA genes of unidentified bacterial isolates were amplified by PCR with 16SUNI-L (5′-AGA GTT TGA TCA TGG CTC AG-3′) as the forward primer and 16SUNI-R (5′-GTG TGA CGG GCG GTG TGT AC-3′) as the reverse primer (Jena Bioscience GmbH, Jena, Germany) to generate approximately 1,400-bp amplicon as published by Kuhnert et al. [13]. PCR products were analyzed by agarose gel electrophoresis, ethidium bromide staining, and visualization under UV light. Bands were cut out, and DNA was purified using a Gel Extraction Kit (Qiagen, Hilden, Germany) according to the manufacturer's recommendations. Cycle sequencing of partial 16S rRNA genes was done in both directions by using forward and reverse amplification primers with a BigDye Terminator Version 1.1 Cycle Sequencing Kit (Applied Biosystems, Darmstadt, Germany) according to the recommendations of the manufacturer. Sequencing products were analyzed with an ABI Prism 3130 Genetic Analyzer (Applied Biosystems). Identification of isolates was done by a BLAST search (http://www.ncbi.nlm.nih.gov/blast/) using 16S rRNA gene sequences.

### 2.5. Genotype Characterization by* E. coli* Genotyping Microarray

Sixteen* E. coli* isolates were selected for the genotype characterization using a DNA microarray system. DNA was extracted as described above. DNA concentration was determined spectrophotometrically. Miniaturized* E. coli* oligonucleotide arrays in the ArrayStrip format (Alere Technologies GmbH, Jena, Germany),* E. coli* Genotyping Kit (Kit for DNA-based detection of virulence genes in* E. coli* isolates, Cat. number 205400050) containing gene targets for the identification of virulence genes [14], antimicrobial resistance genes [15], and DNA-based serotyping [16] were used for the genetic characterization of the* E. coli* isolates.

For labeling and biotinylation of the genomic DNA, a site-specific labeling approach was used as published by Monecke and Ehricht [17]. Primer elongation, hybridization, washing, and staining of array strips were described previously by Geue et al. [18]. The array strips were photographed using an Array Mate instrument (Alere Technologies GmbH) and automatically analyzed. After automated spot detection, mean signal intensity (mean) and local background (lbg) were measured for each probe position and values were calculated by the formula value = 1 − mean/lbg. Resulting values below 0.1 were considered negative and above 0.3 were considered positive. Values between 0.1 and 0.3 were regarded as ambiguous. Validation was performed using a collection of sequenced control strains (GeneBank Accession numbers AE005174 (*E. coli* EDL933 O157:H7), FM180568 (*E. coli* E2348/69 O127:H6), U00096 (*E. coli* K-12 MG1655), AP009048 (*E. coli* K-12 W3110), CP000247 (*E. coli* O6:K15:H31), CP001509 (*E. coli* BL21), AE014075 (*E. coli* CFT073), and CP000946 (*E. coli* ATCC 8739)).

## 3. Results and Discussion

Rapid, accurate, and reliable detection and identification of bacterial foodborne pathogens are critical for food safety. The gold standard is bacterial isolation followed by microscopic and biochemical identifications, which is time-consuming and laborious [6].

In recent years, MALDI-TOF MS has been introduced in microbiological routine laboratories, because it provides results within only a few hours. The instruments are still expensive, but reagent costs are low, and identification of bacteria can be largely automated. The bacteria are identified by comparing the obtained mass spectra to those from a reference library [8]. Limitations have been observed for bacteria that require special sample preparation and some very closely related species. The following bacteria were identified only by partial 16S rRNA gene sequencing:* Staphylococcus sciuri*,* Lysinibacillus* spp., and* Macrococcus caseolyticus*.

The highest number of strains was isolated from raw beef, followed by raw chicken meat, seller stool specimens, and hand swabs from sellers. The high number of* Proteus* isolates from chicken meat (78.0%) and beef meat (58.0%) was remarkable. The presence of* Proteus* spp. in the meat samples can obviously be attributed to unhygienic food processing.* Proteus* spp. were isolated and identified by researchers from raw meat and its products in other studies in Egypt [19–23].

Twenty-seven bacterial isolates out of 50 (54.0%) and 8 out of 50 (16.0%) samples of raw beef and chicken meat, respectively, were identified as* E. coli*. In several studies,* E.coli* was isolated in a high percentage from raw meat and unprocessed ready-to-eat products [24–30]. Contamination may occur due to bowel rupture or use of contaminated water during evisceration and slaughtering [31, 32]. However, neither MALDI-TOF MS analyses nor 16S rRNA gene sequencing allows differentiation between* Shigella* spp. and* E. coli*. A total of 16 presumptively identified* E. coli* isolates were characterized for the genoserotypes,* E. coli* virulence associated genes, and antibiotic resistance genes using a DNA microarray. A complete DNA-based assigned serotype was determined only in five* E. coli* isolates. Only serotype O103:H7 could be characterized completely, because the number of O antigens detectable by this method is currently limited. In 10 other isolates,* fliC* genes for H2 (5 isolates), H49 (found twice), and H19, H31, or H38 were detected, respectively. In one isolate, neither the O-antigen nor the* fliC* gene could be found (Table 2). From 68.0% of the stool samples and 24.0% of the hand swabs of meat sellers,* E. coli* was isolated (Table 1). This high prevalence could be attributed to inadequate sanitary conditions and poor general hygiene. Stephan et al. [33] detected* E. coli* verotoxin encoding genes in 3.5% of healthy employees in the meat industry. Numerous* E. coli* virulence associated markers were tested using the oligonucleotide microarrays. The* stx2* gene was found in one of the 16 isolates only. The isolates were characterized as* stx2a* or* stx2c* or* stx2d* subtypes in accordance with the nomenclature published by Lewis [34]. The microarray does not allow more differentiated subtyping. The* ehxA* gene encoding the EHEC hemolysin was detected in the same isolate. The plasmid encoded virulence genes* esp P* (encoding for a serine protease),* saa* (encoding for the STEC autoagglutinating adhesin),* subA* (encoding for a subtilase cytotoxin), and* iss* (increased serum resistance) were also demonstrated in the same isolate. However, intimin genes were not found. A non-LEE-encoded effector protein gene (*espI*) and the gene for enterobactin siderophore receptor/adhesin (*iha*) were also obtained (Table 2). In the other* E. coli* isolates, the genes for fimbria adhesion (*lpfA*, 14 isolates) and* iss* (increased serum resistance, 7 isolates) were demonstrated frequently. The gene for a glutamate-1-semialdehyde aminotransferase (*hemL*, 5 isolates), the* tsh* gene (encoding for a hemoglobin binding protein, 5 isolates), and the gene for an outer membrane siderophore receptor (*iroN*, 4 isolates) were found rarely. The* cba* gene (encoding for a bacteriocin, 2 isolates), the* cma* gene (also encoding for a colicin, 2 isolates), the* cdtB* gene (encoding for a cytolethal distending toxin, 3 isolates), and the* cnf1* gene (encoding for a cytotoxic necrotizing factor type 1, 3 isolates) were amplified in some isolates. Additional genes for major fimbrial subunit proteins (*f17-A* and* f17-G*) were found in 3 isolates. It is noteworthy that isolates belonging to the same serotype were nearly identical regarding the virulence markers (Table 2).

Genes associated with antibiotic resistance were found rarely. Only in 5 of the 16* E. coli* isolates such genes were observed. The* tetA* (encoding for tetracycline resistance protein A) and the* blaTEM* (encoding for a *β*-lactamase class A) were detected in two isolates, respectively. Genes for three *β*-lactamase associated genes (*blaMOX-CMY9*,* blaOXA*, and* blaVIM*) were found in an On.d.:H2* E. coli* isolate (Table 2).

*Klebsiella* spp. were isolated from 3 samples of raw beef (6.0%) and 13 samples (26.0%) of raw chicken meat. A high incidence of* Klebsiella* spp. from raw meat products was reported previously by Gill [22] and Gibbons et al. [23]. The majority of the Egyptian population purchase raw meat from small local butchers. Meat is usually offered for sale in open-air shops without cooling resulting in potentially heavily contaminated products. Our data confirmed that the locally produced and sold meat is of poor bacteriological quality and poses a high risk for consumer health.

*Klebsiella oxytoca* and* Raoultella ornithinolytica* isolates are known to have very similar spectra in MALDI-TOF MS and can potentially be confused with each other. However, this was not further investigated in this study. Other members of the family* Enterobacteriaceae* causing gastroenteritis like* Proteus* spp.,* Citrobacter* spp.,* Klebsiella* spp., and* Enterobacter* spp. were also isolated from the hands of meat sellers (Table 1).* Proteus* spp. have proved to be a public health hazard and may cause infections of the urinary tract and diarrhea [34]. Food handlers may contaminate their hands with bacteria from either their own stool or during handling of the meat by the food they handle [35]. Neither stool nor food samples yielded* Salmonella* or* Shigella* isolates.

In conclusion, our findings clearly demonstrate that different* Enterobacteriaceae* species are common in retail meat. Insufficient awareness about foodborne zoonoses could endanger both retail sellers and consumers. Education of the traditional meat retailer's community in Egypt in terms of the importance of hygienic and sanitary precautions would be an important step towards safer food.

## Figures and Tables

**Table 1:** Identification of *Enterobacteriaceae* in meat and meat handler samples by MALDI-TOF MS.

Identified microorganism	Raw beef meat (*n* = 50)	%	Raw chicken meat (*n* = 50)	%	Total	%	Stool specimens (*n* = 25)	%	Hand swabs (*n* = 25)	%	Total	%
*Citrobacter* spp.	12	24.0	1	2.0	13	13.0	4	16.0	3	12.0	7	14.0
*C. amalonaticus *	0	0	0	0	0	0	1	4.0	1	4.0	2	8.0
*C. freundii *	5	10.0	1	2.0	6	6.0	3	12.0	1	4.0	4	8.0
*C. braakii *	3	6.0	0	0	3	3.0	1	4.0	1	4.0	2	8.0
*C. koseri *	3	6.0	0	0	3	3.0	0	0	0	0	0	0
*C. youngae *	1	2.0	0	0	1	1.0	0	0	0	0	0	0
*Enterobacter cloacae *	0	0	0	0	0	0	2	8.0	0	0	2	4.0
*Escherichia coli *	27	54.0	8	16.0	35	35.0	17	68.0	6	24.0	23	46.0
*Klebsiella* spp.	3	6.0	13	26.0	16	16.0	1	4.0	9	36.0	10	20.0
*K. pneumoniae *	3	6.0	11	22.0	14	14.0	0	0	6	24.0	6	12
*K. oxytoca *	0	0	2	4.0	2	2.0	1	4.0	3	12.0	4	8.0
*Morganella morganii *	16	32.0	2	4.0	18	18.0	0	0	2	8.0	2	4.0
*Proteus *spp.	29	58.0	39	78.0	68	68.0	15	60.0	7	28.0	22	44.0
*P. vulgaris *	22	44.0	0	0	22	22.0	4	16.0	0	0	4	8.0
*P. mirabilis *	6	12.0	39	78.0	45	45.0	11	44.0	7	28.0	18	36.0
*P. penneri *	1	2.0	0	0	1	1.0	0	0	0	0	0	0
*Providencia* spp.	1	2.0	1	2.0	2	2.0	0	0	0	0	0	0
*Providencia stuartii *	0	0	1	2.0	1	1.0	0	0	0	0	0	0
*Raoultella* spp.	5	10.0	2	4.0	7	7.0	0	0	3	12.0	3	6.0
*R. planticola *	5	10.0	0	0	5	5.0	0	0	1	4.0	1	2.0
*R. ornithinolytica *	0	0	2	4.0	2	2.0	0	0	2	8.0	2	4.0
*Serratia liquefaciens *	1	2.0	0	0	1	1.0	0	0	0	0	0	0

Total number of isolates	94		66		160		39		29		68	

**Table 2:** Phylogenetic characteristics and DNA-based serotypes of tested *Escherichia coli* isolates.

Number	Serotype^*^	Virulence marker	Resistance marker
1	O103:H7	*hemL, lpfA, tsh, iroN, iss *	
2	On.d.:H19		*blaTEM *
3	O103:H7	*hemL, lpfA, tsh, iroN, iss *	
4	O103:H7	*lpfA, tsh, iroN, iss *	
5	On.d.:H38	*stx2 (subtype 2a *or* 2c *or* 2d), ehxA, espI, espP, saa, iha, subA, iss *	
6	On.d.:H2	*cba, cdtB, cma, cnf1, f17-G, lpfA, iroN, iss *	
7	On.d.:H31	*lpfA, cdtB, cnf1, f17-A, f17-G *	
8	O103:H7	*hemL, lpfA, tsh, iroN, iss *	*tetA *
9	On.d.:H49	*hemL, lpfA *	
10	On.d.:H2	*lpfA, cba, cdtB, cma, cnf1, f17-G, iro, iss *	
11	O103:H7	*hemL, lpfA, tsh, iroN, iss *	*tetA *
12	On.d.:Hn.d.	*lpfA *	*blaTEM *
13	On.d.:H49	*lpfA *	
14	On.d.:H2	*lpfA, *	
15	On.d.:H2	*lpfA, *	
16	On.d.:H2	*hemL, lpfA *	*blaMOX-CMY9, blaOXA, blaVIM *

^*^n.d.: not detectable by microarray.

**Table 3:** Identification scores for bacteria isolates obtained by Biotyper software (Bruker Daltonik GmbH) for MALDI-TOF MS results. An identification (ID) score >2.30 is regarded as a highly probable species identification; scores 2.0–2.29 indicate secure genus and probable species identification; scores 1.70–1.99 allow probable genus identification and lower scores provide no reliable results.

Bacterial species	Number of strains	ID score 1.70–1.99	ID score 2.0–2.29	ID score >2.30
*Citrobacter amalonaticus *	1		1	
*Citrobacter braakii *	7			7
*Citrobacter freundii *	8		1	7
*Citrobacter koseri *	3			3
*Citrobacter youngae *	3		1	2
*Comamonas kerstersii *	1		1	
*Enterobacter asburiae *	2	1	1	
*Enterobacter cloacae *	5		2	3
*Enterococcus faecalis *	11		1	10
*Enterococcus faecium *	3		2	1
*Enterococcus raffinosus *	1		1	
*Escherichia coli *	38		11	27
*Hafnia alvei *	2		2	
*Klebsiella oxytoca *	2		1	1
*Klebsiella pneumoniae *	17		10	7
*Klebsiella variicola *	2		2	
*Macrococcus caseolyticus *	4	1	3	
*Morganella morganii *	17			17
*Proteus mirabilis *	40		4	36
*Proteus penneri *	6		2	4
*Proteus vulgaris *	45		9	36
*Providencia rettgeri *	1			1
*Providencia stuartii *	1			1
*Pseudomonas putida *	1		1	
*Pseudomonas aeruginosa *	5			5
*Raoultella ornithinolytica *	6	1		5
*Serratia liquefaciens *	1			1
*Staphylococcus aureus *	4	2	1	1
*Viridibacillus *	1	1		
Unidentified bacteria	4			

Total	242	6	57	175
